# Does country risk impact the banking sectors’ non-performing loans? Evidence from BRICS emerging economies

**DOI:** 10.1186/s40854-023-00494-2

**Published:** 2023-05-07

**Authors:** Chafic Saliba, Panteha Farmanesh, Seyed Alireza Athari

**Affiliations:** 1 Department of Business, Girne American University, Kyrenia, Northern Cyprus Turkey; 2 Department of Business, Girne American University, Kyrenia, Northern Cyprus Turkey; 3 Department of Business Administration, Faculty of Economics and Administrative Sciences, Cyprus International University, Nicosia, Northern Cyprus Turkey; 4 Department of Business, Holy Spirit University of Kaslik, Kaslik, Lebanon

**Keywords:** Credit risk, Country risk, BRICS, Emerging markets, Banking sector, Political risk, Quantile regression, E51, E58, G21

## Abstract

This study aims to fill the gap in the literature by specifically investigating the impact of country risk on the credit risk of the banking sectors operating in Brazil, Russia, India, China, and South Africa (BRICS), emerging countries. More specifically, we explore whether the country-specific risks, namely financial, economic, and political risks significantly impact the BRICS banking sectors’ non-performing loans and also probe which risk has the most outstanding effect on credit risk. To do so, we perform panel data analysis using the quantile estimation approach covering the period 2004–2020. The empirical results reveal that the country risk significantly leads to increasing the banking sector’s credit risk and this effect is prominent in the banking sector of countries with a higher degree of non-performing loans (Q.25 = − 0.105, Q.50 = − 0.131, Q.75 = − 0.153, Q.95 = − 0.175). Furthermore, the results underscore that an emerging country’s political, economic, and financial instabilities are strongly associated with increasing the banking sector’s credit risk and a rise in political risk in particular has the most positive prominent impact on the banking sector of countries with a higher degree of non-performing loans (Q.25 = − 0.122, Q.50 = − 0.141, Q.75 = − 0.163, Q.95 = − 0.172). Moreover, the results suggest that, in addition to the banking sector-specific determinants, credit risk is significantly impacted by the financial market development, lending interest rate, and global risk. The results are robust and have significant policy suggestions for many policymakers, bank executives, researchers, and analysts.

## Introduction

As a financial intermediary, the banking system is unquestionably a crucial sector for running any economy, and any banking sector’s performance is crucial for encouraging investments and boosting economic growth (Menicucci and Paolucci 2021; Bucevska and Hadzi Misheva 2017; Athari 2021a, b). Although the banking sector could be a catalyst to promote economic activity, particularly in countries with weak financial markets, it may be a significant driver to impede economic expansion by not controlling increasing risks. Consequently, governments and central banks place a great deal of importance on the banking industry’s stability.

Nevertheless, after the global financial mortgage crisis (2008–2009), bank asset quality deteriorated severely and many financial institutions operating in both emerging and advanced countries experienced a massive increase in non-performing loans (NPLs). Among risk factors, credit risk, which is measured by the level of NPLs, is considered the most critical risk that threatens the overall stability of the banking sector, and a rise in credit risk^1^ could increase insolvency, instability, and crisis in a country’s banking sector, eventually resulting in deteriorating economic growth (Boudriga et al. 2010; Ariffin 2012; Vouldis and Louzis 2018). Moreover, rising credit risk could lead to sluggish economic output in the long term by reducing profits and lending in the banking sector. Therefore, understanding and determining the factors reducing credit risk is essential for banks and policymakers and help avoid bank failure and increase the effectiveness of the banking sector in promoting economic growth. The Basel Committee on Banking Supervision (BCBS) stated that the management of poor credit risk practices is still a major cause of worldwide banking crises (Ariffi 2012).

Over the last three decades, pervasive studies have been conducted to explore determinants that exacerbate credit risk. Empirical studies suggest that both internal and external factors contribute to credit risk. External elements are specific to the country, and internal elements are the factors specific to the banking industry. More specifically, the majority of empirical studies highlight that liquidity, capital regulation, profitability, inefficiency, and income diversification are the significant banking sector-specific or internal determinants (e.g., Imbierowicz and Rauch 2014; Ali et al. 2015; Anastasiou et al. 2019; Kartikasary et al. 2020; Boussaada et al. 2020). Furthermore, macro-level factors such as gross domestic production (GDP), inflation, real exchange rate, lending interest rate, financial market development, and corruption are considered the most significant external factors (e.g., Dimitrios et al. 2016; Bonilla 2012; Nadham and Nahid 2015; Syed and Tripathi 2020).

What about the determinants of the banking sector’s credit risk in the BRICS emerging countries and how does country risk impact the BRICS banking sector’s credit risk? Although there is extensive literature on the determinants of the banking sector’s credit risk, limited empirical research has been conducted to assess these of the banking sector operating in the BRICS region. To the best of our knowledge, there is relatively little evidence on the empirical level of the relationship between country risk (e.g., political, economic, financial) and the banking sector’s credit risk in developed and developing markets in general and the BRICS region in particular. BRICS is a vital union of Brazil, Russia, India, China, and South Africa, and in 2003, Goldman Sachs described this bloc as one of the developing blocs of the world, contributing a substantially important part of world trade and economy in the future (Singh et al. 2022). On the other hand, BRICS countries are also impacted by issues such as increased in inflation, the decline in the oil markets, weakening regimes in some countries, corruption charges, and the influence of the global financial turmoil (Syed and Tripathi 2020). Focusing on the BRICS banking sector, the World Bank (2017) database indicated that the Brazilian banking sector has higher liquidity reserve assets compared to the BRICS region and global countries while the Russian and South African banking sectors have lower liquidity reserve assets, respectively. Additionally, the Brazilian, Indian, and Chinese banking sectors have higher bank Z-scores (less default risk) relative to the BRICS region and global countries. According to the World Bank (2020) database, the global mortgage crisis adversely impacted the BRICS banking sector and significantly increased NPLs. The BRICS banking system experienced a challenging period between 2014 and 2018 in which many banks experienced declining earnings, declining lending, and rising provisioning (Kondratov 2021). Based on these economic settings, critical factors of banking sector credit risk in the BRICS region is an interesting case study and the findings could considerably contribute to the banking literature.

This study makes several contributions. The first is to fill the gap in the literature by providing new empirical evidence on the banking sector of the BRICS emerging countries. Second, it contributes by investigating the effect of country risk on the BRICS banking sector’s credit risk in addition to the conventional factors. Third, this study addresses how the country-specific economic, political, and financial risks impact the banking sectors’ NPLs in the BRICS countries that are exposed to the financial, economic, and political instabilities mainly after the global financial crisis (2008–2009). Fourth, as in previous studies (Athari 2021a, b, 2022a; Irani et al. 2022), we adopt a composite index of the International Country Risk Guide (ICRG) as a comprehensive and novel proxy for determining a country’s vulnerability. Based on previous studies, the ICRG index is inclusive and accurate and can be used to measure countries’ vulnerability to political, financial, and economic risks. The ICRG rating comprises 22 variables in three subcategories of risk, namely, political, financial, and economic. This study uses a novel proxy of the ICRG political risk index scores to measure the political stability level including government stability, investment profile, socio-economic conditions, internal and external conflicts, corruption, the military in politics, democratic accountability, religious tensions, law and order, ethnic tensions, and bureaucracy quality components. Furthermore, it uses the ICRG economic risk index scores to measure the level of economic stability containing the GDP per capita, real GDP growth, annual inflation rate, budget balance (% of GDP), and Current Account (% of GDP) components. We also use the ICRG financial risk index scores to measure the level of financial stability comprising the foreign debt (% of GDP), foreign debt service (% of exports of goods and services), Current Account (% of exports of goods and services), net international liquidity in months, and exchange rate stability components. This study is significant in that it employs the unique dataset and also opens a new debate in the banking literature. Fifth, this study performs panel quantile estimation for the large panel of data between 2004 and 2020 to derive reliable results. In contrast with ordinary least squares (OLS), quantile regression is more robust to non-normal errors, heterogeneity, and outliers. Using the quantile regression provides an opportunity to explore the relationships between variables across a wide spectrum and specifically examine whether the country risk index and sub-indices impact NPL distributions differently at various points.

This study yields some notable highlights. First, the empirical results reveal that the bank-specific variables of profitability, capital regulation, liquidity, and income diversification significantly and negatively impact the BRICS banking sector’s credit risk whereas inefficiency has the opposite effect. This implies that by increasing profitability, capital regulation, liquidity, income diversification, and efficiency, policymakers and bank managers can reduce the banking sector’s credit risk and minimize the negative consequence of credit risk to enhance financial stability and economic growth. Second, country risk significantly impacts the BRICS banking sector’s credit risk, implying that the banking sector experiences higher NPLs via the increasing vulnerability of BRICS countries to domestic risk factors. Specifically, the results revealed that credit risk increases with rising political, economic, and financial risk and an increase in domestic political instability has the most positive prominent effect on the banking sector of environments with a higher degree of NPLs. This indicates that policymakers should be provided more financially, economically, and politically stable environments by focusing on the risk components such as corruption, government instability, internal and external conflicts, the deficit in the government budget, declining inflation, and exchange rate instability to prevent the banking sector’s credit risk from increasing. Lastly, the empirical results reveal that the financial market development, lending interest rate, and global risk are significant drivers of increasing the BRICS banking sector’s credit risk.

The rest of this paper is organized as follows. Sect. “Literature review” presents a review of the literature. The model specification and data are discussed in Sect. “Model specification and data”. The data analysis and results are provided in Sect. “Data analysis and results”. Sects. “Robustness checks” and “Conclusion and policy implications” present the robustness checks and conclusions and policy implications, respectively.

## Literature review

In the literature, it is well documented that banks’ NPLs are impacted by both micro- and macro-level factors. In this section, we review the findings of previous studies and present the significant factors at both micro- and macro-levels. A summary of the literature is presented in Table 1. Since an increase in NPLs could increase financial instability and adversely impact economic activities, determining significant factors of NPLs is crucial for policymakers and bank managers.

**Table 1:** Summary of literature

Authors	Case-study	Main explanatory variables	Methods
Berger and DeYoung (1997)	U.S banks	Capital adequacy ratio, cost efficiency, bank capital, bank size, loan quality	Dynamic paneldata model
Salas and Saurina (2002)	Spanish banks	GDP real growth, inflation rate, interest rate, the unemployment rate	Dynamic panel data model
Khwaja and Mian (2005)	Pakistani banks	Political power, level of corruption, government stability, political stability	Static panel data model
Quagliariello (2007)	Italian banks	GDP growth rate, inflation, unemployment, interest rate	Dynamic paneldata model
Boudriga et al. (2010)	Banks in MENA region	Credit growth rate, capital adequacy ratio, profitability, real GDP growth rate	OLS estimation approach
Khemraj and Pasha (2009)	Guyanese banking sector	Real effective exchange rate, GDP growth, interest rates, unemployment rate, inflation	Static panel data model
Cotugno et al. (2010)	Italian banks	Bank size, gross loans, functional distance, profitability	Dynamic panel data model
Espinoza and Prasad (2010)	Banks in GCC countries	Capital adequacy ratio, efficiency, bank size, net interest margin, credit growth	Dynamic panel data model
Adebola et al. (2011)	Malaysian Islamic banks	GDP growth, interest rate, inflation, the unemployment rate	ARDL approach
Dimitrios et al. (2016)	Greek banks	GDP growth, consumer loans, business loans & mortgages, unemployment, lending rate	Dynamic panel data model
Kauko (2012)	Banks in European countries	Financial stability, credit growth, bank solvency, liquidity, GDP growth, inflation	OLS estimation approach
Bonilla (2012)	Banks in Spain & Italy	Credit growth, wages, inflation, unemployment, GDP	OLS estimation approach
De Bock and Demyanets (2012)	Banks in emerging countries	Bank asset quality, profitability, capital adequacy ratio, ownership structure	Dynamic panel data model
Louzis et al. (2012)	Greek banks	Profitability, inflation, GDP growth, unemployment rate, lending rate	Dynamic panel data model
Akkoc and Vatansever (2013)	Turkish banks	Exchange rate, GNP growth, unemployment, interest rate, inflation	OLS estimation approach
Badar and Javid (2013)	Pakistan banks	Inflation, exchange rate, interest rate, gross domestic product, money supply	Dynamic panel data model
Messai and Jouini (2013)	Banks in Italy, Greece, & Spain	GDP growth, unemployment rate, real interest rate, inflation	Dynamic panel data model
Abid et al. (2014)	Tunisian banks	Profitability, solvency ratio, inefficiency, bank size	Dynamic panel data model
Imbierowicz and Rauch (2014)	U.S banks	Liquidity risk, bank capital, bank size, efficiency	Dynamic panel data model
Prasanna et al. (2014)	Indian banks	GDP growth rate, savings growth rate, interest rate, inflation	Dynamic panel data model
Beck et al. (2015)	Banks across 75 countries	Nominal effective exchange rate, real GDP, interest rate, stock market capitalization	Static and dynamic panel data model
Ali et al. (2015)	Malaysian commercial banks	Liquidity risk, bank capital, bank size, inefficiency, inflation, profitability, sovereign debt, non-interest income	Dynamic panel data model
Mance et al. (2015)	Banks in Croatia	Real GDP, industrial production index, unemployment rate, inflation, interest rate	Quantile regression estimation method
Nadham and Nahid (2015)	Banks in Tanzania	Interest rate, GDP growth, lending activities, inflation, unemployment	OLS estimation approach
Morakinyo and Sibanda (2016)	Banks in MINT countries	Capital adequacy ratio, profitability, liquidity ratio, bank total credit	Static and dynamic panel data model
Umar and Sun (2016)	Chinese banks	Bank liquidity creation, size, profitability, earning volatility, leverage, interbank offered rate, GDP	Static and dynamic panel data model
Gjeci and Marinc (2018)	Banks across 195 countries	Corruption, bank size, bank effectiveness, bank capital	Static panel data model
Khan et al. (2018)	Banks in Pakistan	Exchange rate, inflation, GDP growth rate, unemployment rate, the tax rate	Static panel data model
Anastasiou et al. (2019)	Greek banking sector	Governance, liquidity risk, political stability, corruption, government effectiveness, unemployment rate, bank concentration, bank deposits, profitability, GDP growth	Static panel data model
Kuzucu and Kuzucu (2019)	Banks in emerging and advanced countries	Exchange rate, inflation, unemployment, economic growth, loan size, bank capitalization, foreign direct investment	Dynamic panel data model
Kumar and Kishore (2019)	Banks in UAE	Profitability, bank efficiency, bank capital, income diversification	Static panel data model
Kartikasary et al. (2020)	Indonesian banks	Bank capital, bank size, loans to deposit ratio, profitability, efficiency	Panel pool regression model
Boussaada et al. (2020)	Banks in the MENA region	Liquidity risk, performance, bank capital, size, financial crisis, inflation, liquid assets	Panel Smooth Transition Regression model
Hakimi et al. (2020)	Banks in the MENA region	Corruption, government stability, bank size, bank capital, GDP, inflation, liquid assets	Self-Exciting Threshold Autoregressive model
Rehmana et al. (2020)	Pakistani banks	Corruption, bank size, profitability, capitalization, GDP, income diversification, inflation, lending interest rate	Static panel data model
Khan et al. (2020)	Pakistan banking sector	Profitability, efficiency, bank capital, income diversification	Static panel data model
Syed and Tripathi (2020)	Indian banks	Inflation, unemployment, GDP growth, saving rate	Dynamic panel data model
Jenkins et al. (2021)	Banks in MENA countries	Corruption, bank size, bank capital, efficiency	Quantile regression estimation method
Karadima and Louri (2021)	banks operating in Europe	Economic policy uncertainty, bank concentration, GDP, inflation, profitability	Dynamic panel data model
Mohamad and Jenkins (2021)	Banks in MENA countries	Corruption, bank-specific factors	Static panel data model
Anita et al. (2022)	Banks in SAARC economies	Government budget balance, GDP, sovereign debt, inflation rate, and money supply	Static panel data model
Fakhrunnas et al. (2022)	Indonesian banks	Growth, inflation, exchange rate, interest rate	NARDL
Foglia (2022)	Italian banks	GDP, public debt, unemployment, domestic credit	ARDL
Naili and Lahrichi (2022)	Banks in MENA countries	capital, inefficiency, size, ownership, GDP, unemployment, inflation, sovereign debt	Static and dynamic panel data model

The majority of studies revealed that the NPLs are impacted by micro-level factors including bank size, profitability,^2^ bank capital, cost efficiency, capital structure, liquidity risk, and asset quality. In an influential study, Berger and DeYoung (1997) determined that the capital adequacy ratio negatively affects the NPLs of US commercial banks. Boudriga et al. (2010) revealed that credit growth rate and capital adequacy ratio are positively and negatively related to banks’ NPLs in the Middle East and North African (MENA) countries, respectively. Boudriga et al. (2009) highlighted that NPLs are significantly influenced by micro-level factors such as capital adequacy and bank ownership, and the banks’ NPLs are reduced in countries with strong legal and institutional conditions. Cotugno et al. (2010) found that the NPLs of Italian banks were positively correlated with bank size, gross loans, and functional distance while negatively related to profitability. Espinoza and Prasad (2010) demonstrated that capital adequacy ratio and efficiency negatively affect banks’ NPLs, while bank size, net interest margin, and lagged credit growth positively affect banks’ NPLs in Gulf Cooperation Council (GCC) countries. De Bock and Demyanets (2012) also revealed that profitability, capital adequacy ratio, bank asset quality, and ownership structure are the main determinants that negatively impact banks’ NPLs in developing countries of the Eurozone.

Furthermore, Louzis et al. (2012) established that profitability negatively affects the NPLs of Greek banks. Using dynamic panel data, Abid et al. (2014) revealed that profitability has a negative significant effect on NPLs while inefficiency positively impacts the NPLs of Tunisian banks. Ekanayake and Azeez (2015) indicated that size and inefficiency are positively correlated with NPLs in the banking sector in Sri Lanka. Imbierowicz and Rauch (2014) and Ali  et al. (2015) determined a positive relationship between liquidity risk and NPLs of banks in the US and Malaysia, respectively. Umar and Sun (2016) also revealed a negative nexus between total liquidity creation and NPLs of Chinese banks between 2005 and 2014. Morakinyo and Sibanda (2016) documented that profitability, liquidity ratio, and capital adequacy ratio have a negative impact on NPLs while the total bank credit positively impacts banks’ NPLs in MINT countries (e.g., Mexico, Indonesia, Nigeria, and Turkey). Rachman et al. (2018) concluded that a rise in profitability and income diversification could reduce the NPLs of Indonesian banks. Anastasiou et al. (2019) demonstrated that liquidity risk has a significant positive impact on NPLs. Kartikasary et al. (2020) indicated that profitability negatively impacts NPLs in Indonesian banks, while the loan-to-deposit ratio has a positive effect. However, Kumar and Kishore (2019) revealed that profitability has an insignificant association with the NPLs of banks in the UAE. Furthermore, Boussaada et al. (2020) highlighted that the liquidity risk significantly increased the NPL level of banks in the MENA region between 2004 and 2017. Khan et al. (2020) highlighted that operating efficiency and profitability have a negative and significant impact on NPLs in the banking sector in Pakistan. In addition, capital adequacy and income diversification negatively impact NPLs but the effect is statistically insignificant. Naili and Lahrichi (2022) also established that bank-specific factors including capital, performance, operating inefficiency, size, and ownership concentration impact banks’ NPLs in the MENA economies.

Another strand of literature underscored that macro-level factors significantly impact banks’ NPLs. Numerous studies found that factors including GDP growth, inflation rate, interest rate, and unemployment rate are the most significant macroeconomic factors of NPLs. In the earliest study, Keeton and Morris (1987) revealed that poor economic conditions affect the loan portfolios of banks in the US. Salas and Saurina (2002) found that the real growth in GDP is an important factor in explaining the variation in NPLs of banks in Spain. Quagliariello (2007) determined that a rise in economic growth reduces banks’ NPLs while the inflation rate has the opposite effect. Khemraj and Pasha (2009) highlighted that GDP growth has a significant negative impact on NPLs in the Guyanese banking sector. Their findings also indicated that banks that charge relatively higher interest rates and lend excessively are more likely to incur higher levels of NPLs. By examining the effect of macro-level factors on NPLs of Malaysian Islamic banking, Adebola et al. (2011) documented that interest rate has a significant positive long-run impact on NPLs. Dimitrios et al. (2016) found that the real GDP growth rate negatively affects the NPLs of Greek banks while the unemployment rate has a positive effect.

Similarly, Bonilla (2012) showed that unemployment and GDP have statistically significant effects on the NPLs of banks in Spain and Italy, while credit growth and inflation have a statistically insignificant effect. Badar and Javid (2013) revealed the pair-wise long-run relationship between banks’ NPLs with money supply and interest rate in Pakistan. Additionally, the Granger causality test indicated that the inflation and exchange rate Granger caused NPLs. Moreover, there is a weak short-run relationship between inflation and exchange rate with NPLs. Messai and Jouini (2013) established that banks’ NPLs are negatively impacted by the growth rate of GDP in Italy, Greece, and Spain while positively impacted by the real interest rate and unemployment rate. Selma and Fathi (2013) suggested that the GDP growth rate negatively affects the NPLs of banks in Greece, Spain, and Italy, while the unemployment rate has a positive effect. Skarica (2013) also demonstrated that GDP has a negative significant effect on NPLs in central and eastern European countries whereas unemployment and inflation have a positive effect. Prasanna (2014) found that higher GDP growth is negatively associated with NPLs of Indian banks whereas the higher interest and inflation rates positively impact NPLs. Mance et al. (2015) revealed that NPLs of banks in Croatia are negatively affected by real GDP and industrial production index while unemployment impacts positively. Nadham and Nahid (2015) established that interest rate has a positive relationship with banks’ NPLs in Tanzania whereas GDP growth has a negative effect. Recently, Anastasiou et al. (2019) determined that the governance system has a significantly negative impact on NPLs of the banking sector in Greece. Syed and Tripathi (2020) showed that unemployment has a positive relationship with banks’ NPLs in the BRICS countries whereas GDP growth and financial soundness are negatively impacted. The findings also revealed that savings by household and inflation rate positively impact NPLs. Karadima and Louri (2021) identified that economic policy uncertainty positively impacts the NPLs of banks operating in France, Germany, Italy, and Spain; however, the extent of the effect is significantly controlled by bank concentration. Anita et al. (2022) documented that government budget balance positively impacts banks’ NPLs in South Asian Association for Regional Cooperation (SAARC) economies while GDP, sovereign debt, inflation rate, and money supply have a negative impact on banks’ NPLs. Similarly, Fakhrunnas et al. (2022) revealed an asymmetric nexus between macroeconomic factors and the banks’ NPLs in Indonesia both before and during the COVID-19 pandemic. Foglia (2022) underlined that GDP and public debt negatively impact NPLs in the Italian banking system whereas the unemployment rate and domestic credit have the opposite effect. Naili and Lahrichi (2022) also established that GDP growth, unemployment, inflation, and sovereign debt significantly impact banks’ NPLs in the MENA economies.

More specifically, several studies emphasized that in addition to the aforementioned factors mentioned, domestic political risk significantly impacts banks’ NPLs. For instance, Khwaja and Mian (2005) found that political power and corruption practices are significant drivers of high degrees of NPLs of banks in Pakistan. Kaufmann and Kraay (2007) revealed that both political stability and governance factors negatively impact banks’ NPLs. Kastrati (2011) determined that the implementation of the rule of law is an important factor in explaining the NPLs in transition economies. Gjeci and Marinc (2018) highlighted a positive and statistically significant relationship between corruption and NPLs of banks globally. Anastasiou et al. (2019) also revealed that the governance system negatively impacted NPLs of the Greek banking sector between 1996 and 2016. Hakimi et al. (2020) examined the relationship between government stability, corruption, and NPLs of banks in the MENA region and found that the banks’ NPLs are negatively impacted by decreasing corruption and increasing government stability and the effect is significant only when attaining a certain level; up to that point, the impact is insignificant. Rehmana et al. (2020) indicated that higher control of corruption would lower the NPLs of banks in Pakistan. Recently, Jenkins et al. (2021) revealed that corruption significantly exacerbates the problem of NPLs of banks in MENA countries and does not affect all banks at the same level. Mohamad and Jenkins (2021) also highlighted that corruption positively affects banks’ NPLs in the MENA region.

Moreover, several studies have examined the effect of domestic financial risk on banks’ NPLs. For instance, Khemraj and Pasha (2009) highlighted that the increasing real effective exchange rate has a significant positive impact on NPLs in the Guyanese banking sector. Through an investigation of the relationship between the exchange rate and NPLs for Turkish banks, Akkoc and Vatansever (2013) revealed that the exchange rate positively impacts banks’ NPLs. Beck et al. (2015) established that the rise in domestic financial instability through increasing nominal effective exchange rate positively affects banks’ NPLs in 75 countries. Merz (2017) also demonstrated that the exchange rate volatility has a statistically significant positive impact on NPLs in 62 countries. Khan et al. (2018) revealed that the increasing real exchange rate leads to increasing NPLs of banks in Pakistan. The majority of the cited studies highlighted that the micro- and macro-level factors impact the NPLs of banks. In the literature, a limited number of studies deeply examine the effect of country risk, in particular, economic, financial, and political risks on banks’ NPLs. Moreover, some studies investigated this relationship for the banking sector in general and in the context of BRICS countries in particular. Therefore, the current study addresses the gaps in the related literature by examining the impact of country-specific risks including the political, financial, and economic risks on the NPLs of the banking sector operating in BRICS between 2004 and 2020.

## Model specification and data

### BRICS banking system

The characteristics of the BRICS banking sector are presented in Table 2. Focusing on the ratios related to the banking industry, Brazilian and Russian banks have greater capital ratios than other BRICS nations, with average values of 10.08 and 11.55, respectively. The Brazilian banking industry has the largest liquidity reserve assets, with an average value of 25.04, compared to the BRICS area and globally, which have average values of 12.91 and 22.45, respectively. In contrast, the banking sectors of South Africa and Russia, with averages of 3.48 and 10.20, respectively, have the lowest liquidity reserves assets.

**Table 2:** Characteristics of the BRICS banking sector

Banking sector-specific ratios	% Average (2004–2017)						
	Brazil	Russia	India	China	South Africa	BRICS Region	Global (N = 214)
Bank capital to asset ratio	10.08	11.55	6.87	6.17	7.48	8.43	10.17
Bank liquid reserves to bank assets ratio	25.04	10.20	–	–	3.48	12.91	22.45
Bank non-performing loans to total gross loans ratio	3.34	6.11	4.65	3.68	3.33	4.22	6.32
Domestic credit to private sector by banks ratio (%GDP)	51.64	44.72	47.93	128.09	145.92	83.66	52.66
Five-bank asset concentration	72.94	41.33	41.82	68.03	99.13	64.65	80.01
Bank cost to income ratio	58.88	72.92	47.37	37.36	57.48	54.8	56.33
Bank regulatory capital to risk-weighted assets	17.49	15.10	13.02	10.77	14.21	14.12	17.13
Bank Z-score	15.71	7.33	16.36	18.66	13.64	14.34	13.70

Bank capital to asset ratio measured as the bank capital and reserves divided to total assets; Bank liquid reserves to bank assets ratio measured as the domestic currency holdings and deposits with the monetary authorities to claims on other governments, nonfinancial public enterprises, the private sector, and their banking institutions; Bank non-performing loans to total gross loans ratio measured as the value of nonperforming loans divided by the total value of the loan portfolio; Domestic credit to private sector by banks ratio refers to the financial resources provided to the private sector by other depository corporations; Five-bank asset concentration measured as the assets of five largest banks as a share of total commercial banking assets; Bank cost to income ratio measured as the operating expenses of a bank as a share of sum of net-interest revenue and other operating income; Bank regulatory capital to risk-weighted assets measured as the capital adequacy of deposit takers; Bank Z-score captures the probability of default of a country’s commercial banking system

*Source*: World Bank (Global financial development and World development indicators)

Moreover, with an average value of 3.33, 3.34, and 3.68, South African, Brazilian, and Chinese banking sectors respectively have lower credit risk relative to the BRICS region, which has an average ratio of 4.22. Remarkably, banking sectors in the region have experienced lower credit risk relative to the global countries with an average ratio of 6.32. Furthermore, Table 2 shows that with an average value of 68.03, 72.94, and 99.13, respectively, Chinese, Brazilian, and South African banking sectors have higher market concentration relative to the BRICS region, which has an average ratio of 64.65. With an average value of 99.13, only the South African banking sector has the highest market concentration relative to the global countries, which have an average ratio of 80.01. Additionally, compared to the BRICS area and countries worldwide, which have average ratios of 14.34 and 13.70, respectively, the Brazilian, Indian, and Chinese banking sectors have higher bank Z-scores of 15.71, 16.36, and 18.66, respectively.

### Data

Owing to the importance of the banking industry in the BRICS emerging countries, this study concentrated on the banking sectors operating in these countries between 2004 and 2020. This time frame was selected because of data availability and to prevent missing observations. Annual data for the country-level and banking sector-specific variables were obtained from the World Bank database and the websites of the Central Banks.^3^ Additionally, we acquired data from the PRS group for the ICRG country risk index and its sub-indices, namely political, economic, and financial risk.^4^ The annual global economic policy uncertainty index scores were also gathered from the policy uncertainty website.^5^

### Variable specification

Definitions, expected signs, and sources of the variables used in this study are listed in Table 3. Following earlier empirical studies, this study uses the ratio of the value of non-performing loans to the total value of the loan portfolio (NPL/TL) as of measurement of non-performing loans. Additionally, consistent with earlier findings (e.g., Beck et al. 2015; Ouhibi and Hammami 2015), this study decomposes the determinants at the banking sector-specific (micro) and country (macro)-level. The banking sector-specific determinants include liquidity measured by bank liquid reserves to bank assets ratio (LIQ/TA), capital regulation measured by bank regulatory capital to risk-weighted assets ratio (REQ/RWA), profitability measured by return on assets ratio (ROA), inefficiency measured by bank cost to income ratio (OC/TA), and income diversification measured by bank non-interest income to total income (NI/TI).

**Table 3:** Definitions and sources of variables

Variables	Definitions	Signs	Sources
*Dependent variable*			
Non-performing loans	Value of non-performing loans divided by the total value of the loan portfolio (NPL/TL)		World Bank
*Independent variables*			
Banking sector-specific variables			
Liquidity	Bank liquid reserves to bank assets ratio (%) (LIQ/TA)	–	
Capital regulation	Bank regulatory capital to risk-weighted assets (%) (REQ/RWA); Bank capital to total assets (%) (C/TA)	–	World Bank, Central Banks
Profitability	Bank return on assets (ROA); Bank return on equity (ROE)	–	
Inefficiency	Bank cost to income ratio (%) (C/I); bank overhead costs to total assets (%) (OC/TA)	+	
Income diversification	Bank non-interest income to total income (%) (NI/TI)	–	
Country and global-level variables			
Country risk index	Country risk is an index using the proxy of the ICRG country risk index (CRI). A country’s risk score is between 0 to 100, which 0 indicating the highest risk and 100 as the lowest risk	±	www.prsgroup.com
Political risk index	Political risk is an index (PRI) including government stability, socio-economic conditions, investment profile, internal conflict, external conflict, corruption, military in politics, religious tensions, law and order, ethnic tensions, democratic accountability, and bureaucracy quality. The score range is from 0 to 100, which 0 indicating the highest risk and 100 as the lowest risk	±	
Economic risk index	Economic risk is an index (ERI) containing the GDP per head, real GDP growth, annual inflation rate, budget balance (Percent of GDP), and current Account (percent of GDP). An economic risk score is between 0 to 50, which 0 indicating the highest risk and 50 as the lowest risk	±	
Financial risk index	Financial risk is an index (FRI) containing the foreign debt (Percent of GDP), foreign debt service (percentage of exports of goods and services), Current Account (percentage of exports of goods and services), net international liquidity in months, and exchange rate stability. A financial risk score is between 0 to 50, with 0 indicating the highest risk and 50 as the lowest risk	±	
Financial market development	Domestic credit provided by the banking sector to GDP (%) (DC/GDP)	+	World Bank
Lending interest rate	The lending rate is the bank rate that usually meets the short- and medium-term financing needs of the private sector (LIR)	+	
Global risk	The annual Global Economic Policy Uncertainty index (GR) is based on a GDP-weighted average of national EPU indices for 20 foreign countries. A higher GR score indicates a higher global risk	+	www.policyuncertainty.com

Table 3 describes all using variables that are used in the econometric model

For the country level, the proxy of the ICRG country risk index^6^ (CRI) (0–100) and its sub-indices scores containing the political risk (PRI) (0–100), economic risk (ERI) (0–50), and financial risk (FRI) (0–50) are used to assess a country’s vulnerability to the domestic risks. We also include the domestic credit-to-GDP ratio provided by the banking sector (DC/GDP) as a proxy of financial market development and the bank offering rate for the short- and medium-term financing needs (LIR) as a measurement of lending interest rate. Moreover, the global economic policy uncertainty index score^7^ (GR) is used as a proxy for assessing global risk.

### Model specification and methodology

To reduce the impact of outliers, this study winsorized the variables for each year from the top and bottom 1% before completing estimations (e.g., Athari and Bahreini 2021; Athari 2022b, c). However, panel data methodology was used to reduce heterogeneity and multicollinearity concerns and also increase the effectiveness of estimations, building on the work of Baltagi et al. (2005) and Hsiao and Tseng (2014). Following Jenkins et al. (2021), the current study employs quantile regression to describe the link at various points in the conditional distribution of the dependent variable for model estimation. Quantiles are cut points that divide the range of a probability distribution into narrower intervals with the same probability. This unique feature of quantile regression offers the opportunity to explore whether country risk impacts NPL distributions differently at various points.

Similarly, quantile regression is more resistant to non-normal errors and outliers than OLS, which may be ineffective if the errors are significantly non-normal (Liu et al. 2013). Additionally, quantile regression offers a fuller characterization of the data by enabling us to consider a covariate’s impact on the entire distribution of *y* rather than just its conditional mean. Furthermore, this method provides a highly comprehensive analysis of the relationships between variables across a wide spectrum. In contrast to the mean regression, quantile regression does not require data to follow a specific distribution, making it possible to estimate a variety of effects based on the quantiles of the response variables. A quantile regression method can also manage heterogeneity when dealing with data gathered from different sources, locations, and times (Qin and Reyes 2011; Qin 2012). To check the endogeneity issue in the estimation models, the Generalized Method of Moments (GMM)-SYS dynamic panel data approach and Granger causality are performed, and the results are presented in the robustness checks. Overall, the quantile panel estimation results are robust, and the estimation models are not suffering from the endogeneity problem.

If the dependent variable is a linear function of the independent variables ($y=\beta {X}^{^{\prime}}+\upvarepsilon$), the quantile regression estimator for quantile *q* minimizes the objective function as

1 $$\mathrm{Q}\left({\upbeta }_{\mathrm{q}}\right)= \sum_{\mathrm{i}: {\mathrm{y}}_{\mathrm{i}}\ge {\mathrm{X}}_{\mathrm{i}}^{{^{\prime}}}{\varvec{\upbeta}}}^{\mathrm{N}}\mathrm{q}\mid {\mathrm{y}}_{\mathrm{i}}-{\mathrm{X}}_{\mathrm{i}}^{{^{\prime}}}{\upbeta }_{\mathrm{q}}\mid +\sum_{\mathrm{i}: {\mathrm{y}}_{\mathrm{i}}< {\mathrm{X}}_{\mathrm{i}}^{{^{\prime}}}{\varvec{\upbeta}}}^{\mathrm{N}}\left(1-\mathrm{q}\right)\mid {\mathrm{y}}_{\mathrm{i}}-{\mathrm{X}}_{\mathrm{i}}^{{^{\prime}}}{\upbeta }_{\mathrm{q}}\mid$$

Thus, $\acute{\beta} \left( p \right)$, for any quartile level (p) between 0 and 1 is considered the *p*th regression quantile, this will lead to the sum of the weighted absolute residuals being diminished (Koenker and Bassett Jr. 1978; Qin et al. 2010).

The usage of the practical form specified below tests the determinants of the banking sector’s NPLs.

$$Non\;performing\;loans = f(Banking\;sector\;specific,\;country\& global\;levels)$$

The expanded aforementioned practical form is presented in Eq. (1).

2 $$\begin{aligned}{NPL/TL}_{it \, } & = { \alpha }_{0}+{\alpha }_{1}{LIQ/TA}_{it}+ \, {\alpha }_{2}{REQ/RWA}_{it}+ \, {\alpha }_{3}{ROA}_{it}+ \, {\alpha }_{4}{C/I}_{it}\\ & \quad + \, {\alpha }_{5}{NI/TI}_{it}+ \, {\alpha }_{6}{CRI}_{it}+{\alpha }_{7}{DC/GDP}_{it}\\ & \quad + \, {{\alpha }_{8}{LIR}_{it}+ \, {\alpha }_{9}{GR}_{it}+\varepsilon }_{it}\\ \end{aligned}$$

where _it_ represents country and time, respectively. ε_it_ is an independent error term. NPL/TL is a banking sector non-performing loan. For the banking sector-specific variables, liquidity (LIQ/TA), capital regulation (REQ/RWA), profitability (ROA), inefficiency (C/I), and income diversification (NI/TI) are employed. Moreover, the country risk index (CRI), financial market development (DC/GDP), lending interest rate (LIR), and global risk (GR) are used for the country and global-level variables.

In addition, the effect of country-specific risks in particular political, economic, and financial risk indices on the banking sectors’ NPLs is examined using Eq. (2).

3 $$\begin{aligned} NPL/TL_{{it{\kern 1pt} }} & = \alpha _{0} + \alpha _{1} LIQ/TA_{{it}} + \,\alpha _{2} REQ/RWA_{{it}} + \,\alpha _{3} ROA_{{it}} + \,\alpha _{4} C/I_{{it}} \\ & \quad + \,\alpha _{5} NI/TI_{{it}} + \alpha _{6} PRI_{{it}} + \alpha _{7} ERI_{{it}} + \alpha _{8} FRI_{{it}} \\ & \quad+ \alpha _{9} DC/GDP_{{it}} + \,\alpha _{10} LIR_{{it}} + \,\alpha _{11} GR_{{it}} + \varepsilon _{{it}} \\ \end{aligned}$$

where PRI, ERI, and FRI is the political, economic, and financial risk index, respectively.

## Data analysis and results

### Descriptive statistics

A summary of the descriptive statistics, including the average and the median values for all of the variables, for both the individual and pooled countries under investigation between 2004 and 2020 is provided in Table 4. Panel (A) indicates that China and Russia with a median value of 1.747 and 6.731 have the lowest and highest NPLs, respectively. Similarly, with a median value of 44.014, 64.368, and 60.734, Russia has the highest liquidity, inefficiency, and income diversification ratios, respectively, whereas China has the lowest with a median value of 1.017, 0.964, 37.179, and 15.427. For the country-level variables, Panel (B) shows that Russia and China have the lowest financial market development and lending interest rate with a median value of 45.257 and 5.581, respectively. Additionally, with the median value of 74.333 and 69.385, China and South Africa are the least and most vulnerable countries, respectively. The median value of 124.121 for global economic policy uncertainty also reveals a high level of policy uncertainty on the global economic level, and this result is observed across the entire study.

**Table 4:** Descriptive summary (2004–2020)

Sample country	Non-performing loans (NPL/TL)		Liquidity (LIQ/TA)		Capital regulation (REQ/RWA)		Profitability (ROA)		Inefficiency (C/I)		Income diversification (NI/TI)	
	Mean	Median	Mean	Median	Mean	Median	Mean	Median	Mean	Median	Mean	Median
*Panel (A): Descriptive summary of banking sector variables*												
Brazil	3.244	3.111	19.001	17.002	63.882	65.011	1.545	1.588	58.868	57.838	34.832	32.414
Russia	6.687	6.731	30.012	44.014	34.059	31.003	1.138	1.003	72.925	64.368	63.863	60.734
India	5.393	4.346	1.001	1.034	20.294	18.081	0.769	0.979	47.375	46.523	31.921	30.849
China	3.241	1.747	1.011	1.017	29.471	27.002	0.952	0.964	37.359	37.179	16.615	15.427
South Africa	3.466	3.641	32.022	32.019	37.706	41.001	1.162	1.161	57.477	57.743	46.534	46.817
Total	4.406	3.467	16.601	10.001	37.082	36.066	1.113	1.019	54.801	54.717	38.752	35.776

Sample country	Financial market development (DC/GDP)		Lending interest rate (LIR)		Country risk index (CRI)		Global economic policy uncertainty (GR)	
	Mean	Median	Mean	Median	Mean	Median	Mean	Median
*Panel (B): Descriptive summary of country-level variables*								
Brazil	53.868	59.484	42.638	43.717	69.651	69.792	141.361	124.121
Russia	44.831	45.257	10.728	10.561	71.799	71.938	141.361	124.121
India	48.631	50.126	10.487	10.251	69.411	69.729	141.361	124.121
China	134.946	128.918	5.379	5.581	74.673	74.333	141.361	124.121
South Africa	69.096	67.585	10.381	10.125	69.686	69.385	141.361	124.121
Total	70.274	59.967	15.922	10.292	71.044	70.865	141.361	124.121

Sample country	Political risk index (PRI)		Economic risk index (ERI)		Financial risk index (FRI)	
	Mean	Median	Mean	Median	Mean	Median
*Panel (C): Descriptive summary of country risk sub-index variables*						
Brazil	65.719	66.208	35.188	35.792	38.411	38.938
Russia	61.333	60.729	38.617	39.625	43.648	44.333
India	61.331	61.458	34.526	35.167	42.979	43.146
China	62.031	61.021	40.227	40.001	47.135	47.479
South Africa	66.539	66.354	34.339	33.667	38.448	38.542
Total	63.391	63.396	36.579	36.396	42.124	42.396

This table shows the descriptive statistics of the explanatory variables in the regressions

Moreover, Panel (C) presents a descriptive summary of country-specific risks indicating that, with a median value of 66.354, South Africa has the least politically vulnerable environment. China has the least economically and financially vulnerable environment, with a median value of 40.001 and 47.479, respectively. As reported in Table 4, the median value of the political and economic risk index is 63.396 and 36.396, respectively, indicating that BRICS countries experience moderate levels of both political and economic risks. However, with a median value of 42.396, BRICS countries are positioned at a very low level in terms of financial risk.^8^

**Fig. 1:**
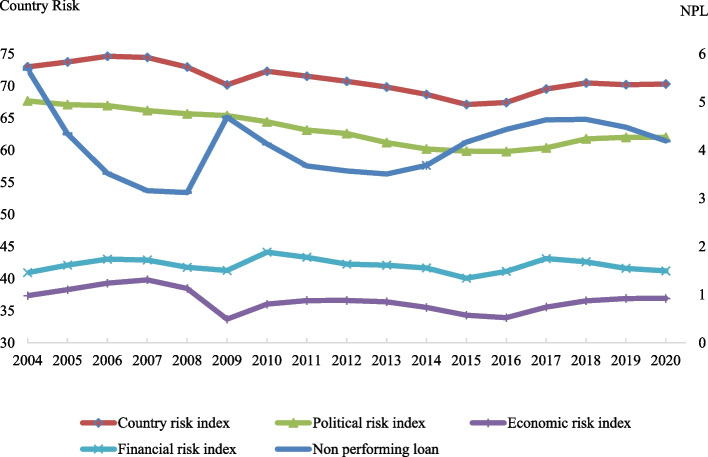
Time series plot of country risk indices and non-performing loans

The time series plot of country risk indices and NPLs from 2004 to 2020 is illustrated in Fig. 1. As demonstrated, there is a downward trend in the country risk index, indicating a rise in the countries’ vulnerability to political, economic, and financial risks in the BRICS countries over the investigated period. Additionally, BRICS banking sectors’ NPLs significantly increased during the global financial crisis (2008–2009). However, after the post-global crisis period, NPLs declined except for the period between 2014 and 2018 owing to risks associated with regulation and policy, and also excessive inflation (Moudud-Ul-Huq 2020). Several industries such as the steel, textile, telecom, and infrastructure industries all experienced severe financial and operational stress as a result, which ultimately had a negative effect on bank profitability and financial stability. Balasubramanian et al. (2019) argued that since the bulk of these financial institutions lacked a comprehensive framework or regulations and an early warning system for evaluating the state of the economy, there was an increase in NPLs in the BRICS nations. Additionally, the lack of a prompt investigative system for examining the intent and business justification of defaulting borrowers as well as insufficient credit appraisal mechanisms contributed to an increase in NPLs.

The Pearson correlation matrix and variance inflation factors (VIF) for using variables are presented in Table 5. The results indicate the suggested model is significantly free from the problem of multicollinearity except for the country-specific risk sub-indices. Thus, we test the effect of political, economic, and financial risk indices separately to avoid the multicollinearity problem.

**Table 5:** Pearson correlation matrix and variance inflation factors (VIF)

	LIQ/TA	REQ/RWA	ROA	C/I	NI/TI	PRI	ERI	FRI	CRI	DC/GDP	LIR	GR	VIF
LIQ/TA	1.000												1.10
REQ/RWA	0.013	1.000											1.15
ROA	0.017	0.119*	1.000										1.22
C/I	0.335*	0.109*	0.096	1.000									1.09
NI/TI	0.235*	0.172	0.076	0.194*	1.000								1.15
PRI	− 0.027	0.253*	0.109*	0.102	− 0.036	1.000							1.13
ERI	− 0.249*	− 0.121	0.281*	− 0.169	− 0.172	0.257*	1.000						1.06
FRI	− 0.138*	− 0.161*	− 0.054	− 0.135*	− 0.251*	− 0.231*	0.348*	1.000					1.14
CRI	− 0.163*	0.007	0.192*	− 0.113	− 0.228*	0.522*	0.662*	0.549*	1.000				1.17
DC/GDP	− 0.287*	− 0.194*	− 0.120*	− 0.093*	− 0.127*	− 0.221*	0.316*	0.292*	0.183*	1.000			1.07
LIR	0.113*	0.177*	0.135*	0.179*	− 0.019	0.192*	− 0.238*	− 0.106*	− 0.246*	− 0.182*	1.000		1.18
GR	0.202*	− 0.014	− 0.164*	− 0.023	0.060	− 0.233*	− 0.191*	− 0.006	− 0.247*	0.110*	− 0.106	1.000	1.22

*Statistically significant at 1%

### Empirical results

Before the estimation of equations, the stationarity test is performed to prevent a spurious estimation. Similar to the recent studies by Athari (2021a; b), the panel unit root techniques suggested by Levin et al. (2002) and Im et al. (2003) were used. The results are reported in Table 6 and indicate that the investigated factors are stationary after the first difference for both trend and cross-sectional dependence alternatives.

**Table 6:** Unit root test results

Variables	Panel (A): Levin–Lin–Chu (2002)		Panel (B): Im–Pesaran–Shin (2003)	
	With trend	With cross-sectional dependence	With trend	With cross-sectional dependence
Non-performing loans	6.563*	− 11.321*	− 2.121**	− 10.523*
Profitability	− 12.244*	− 10.553*	− 5.554*	− 5.415*
Capital regulation	− 7.351*	− 6.753*	− 6.332*	− 7.254*
Liquidity	− 5.241*	− 5.635*	− 12.554*	− 6.346*
Inefficiency	− 11.231*	− 6.566*	− 7.433*	− 9.431*
Income diversification	− 17.533*	− 11.328*	− 6.243*	− 1.977***
Country risk index	− 10.436*	− 6.257*	− 5.653*	− 5.568*
Political risk index	− 5.533*	− 7.588*	− 6.268*	− 5.433*
Economic risk index	− 6.257*	− 6.438*	− 11.463*	− 7.436*
Financial risk index	− 10.352*	− 5.478*	− 8.577*	− 10.411*
Financial market development	− 11.127*	− 6.244*	− 3.435**	− 3.435*
Lending interest rate	− 13.251*	− 4.332*	− 4.525*	− 2.328*
Global risk	− 9.425*	− 6.553*	− 6.356*	− 7.326*

Table 6 shows the panel unit root test results of investigated variables. The null hypothesis of Levin–Lin–Chu (LLC) and Im–Pesaran–Shin (IPS) unit root test is panels contain unit roots. The symbols *, **, and *** denote statistical significance at the 1%, 5%, and 10% levels, correspondingly

The estimation results for Eq. (2) for quantiles Q.25, Q.50, Q.75, and Q.95 are reported in Table 7. The results reveal that the coefficient of profitability (ROA) is negative and significant, implying that banks with more profitability have lower NPLs, which supports previous studies (e.g., Cotugno et al. 2010; Louzis et al. 2012; Morakinyo and Sibanda 2016; Rachman et al. 2018). In particular, the results demonstrate that a rise in profitability has the most negative prominent effect on the banking sector of BRICS countries with a higher degree of NPLs (Q.95 = − 0.728). This is in line with previous studies by Salas and Saurina (2002), Rajan and Dhal (2003), and Athari (2021a, b), who established that larger banks are more profitable and are likely to be more diversified, have advanced technologies, and are more involved with risk management.

**Table 7:** The impact of country risk rating on the banking sector’s credit risk (2004–2020)

Independent Variables	Quantile estimated coefficients			
	Q.25	Q.50	Q.75	Q.95
*Banking sector-specific variables*				
Profitability	− 0.413	− 0.444*	− 0.743*	− 0.728*
	(0.136)	(0.002)	(0.008)	(0.000)
Capital regulation	− 0.046**	− 0.036	− 0.042**	− 0.028*
	(0.021)	(0.241)	(0.032)	(0.001)
Liquidity	− 0.081*	− 0.052*	− 0.080***	− 0.035
	(0.002)	(0.000)	(0.086)	(0.332)
Inefficiency	0.185	0.044	0.026*	0.089***
	(0.119)	(0.158)	(0.002)	(0.094)
Income diversification	− 0.247	− 0.539***	− 0.336***	− 0.453
	(0.151)	(0.094)	(0.093)	(0.256)
*Country and global-level variables*				
Country risk index	− 0.105**	− 0.131*	− 0.153**	− 0.175***
	(0.026)	(0.003)	(0.021)	(0.064)
Financial market development	0.013	0.016**	0.012*	0.022***
	(0.254)	(0.044)	(0.000)	(0.064)
Lending interest rate	0.073	0.182**	0.135**	0.057*
	(0.321)	(0.034)	(0.038)	(0.002)
Global risk	0.031**	0.036*	0.034*	0.022***
	(0.021)	(0.000)	(0.001)	(0.068)
TC dummies	YES	YES	YES	YES

Table 7 shows the effect of the country risk index on the BRICS banking sector’s credit risk. Profitability is the bank return on assets; Capital regulation is the bank regulatory capital to risk-weighted assets; Liquidity is the bank liquid reserves to bank assets ratio; Inefficiency is the bank cost to income ratio; Income diversification is the bank non-interest income to total income; Country risk index is the ICRG country risk rating; Financial market development is the domestic credit provided by the banking sector to GDP; Lending interest rate is the bank rate that usually meets the short- and medium-term financing needs of the private sector; Global risk is global economic policy uncertainty. TC dummies are time and country dummies. The 25th, 50th, 75th, and 95th percentiles of the NPL rate are reported. Numbers in parentheses for each column denote the *P* values. *, **, and *** denote the significance level at 1%, 5%, and 10%, respectively

Moreover, the results indicate that the coefficients of capital regulation (REQ/RWA) and liquidity (LIQ/TA) are negative and significant, and increasing them results in declining NPLs. Similarly, previous studies (e.g., Imbierowicz and Rauch 2014; Morakinyo and Sibanda 2016; Boussaada et al. 2020) confirmed that credit risk is negatively impacted by rising capital regulation and liquidity. Based on the “moral hazard” hypothesis, banks that stockpile a relatively smaller amount of bank capital are inclined to increase the size and riskiness of their loan portfolio, resulting in more bad loans and an increase in NPLs (Zhang et al. 2016; Kuzucu and Kuzucu 2019). The results of the panel quantile regression also document a positive relationship between a banking sector’s inefficiency (C/I) and NPLs, but it is only significant in the 0.75 (Q.75 = 0.026) and 0.95 quantiles (Q.95 = 0.089). This result is consistent with the findings of Abid et al. (2014) and Ali et al. (2015), and also supports the “bad management” hypothesis, suggesting that banks with poor cost management are probable to have high credit risk. As reported in Table 7, the coefficient of income diversification (NI/TI) is negative but only significant in the 0.50 (Q.50 = − 0.539) and 0.75 (Q.75 = − 0.336) quantiles. Consequently, this is consistent with a “diversification” hypothesis and also Kumar and Kishore’s (2019) research, implying that banks with increasing non-interest income could curb credit risk. Overall, the results suggest that banking sector-specific variables are significant predictors of NPLs in BRICS countries.

On the other hand, the assessment of the country and global-level variables reveals that the coefficient of country risk (CRI) is statistically negatively significant for several quantiles. This implies that banking sectors’ NPLs react significantly to the BRICS country’s level of vulnerability to risk factors, with banks’ NPLs increasing (or decreasing) as vulnerability increases (or decreases). Our results support the findings of previous studies (e.g., Quagliariello 2007; Beck et al. 2015; Khan et al. 2018; Syed and Tripathi 2020; Jenkins et al. 2021), which highlighted that environmental characteristics matter and banks’ NPLs increase with the rising vulnerability of countries to the economic (e.g., rising inflation, declining GDP), financial (e.g., exchange rate volatility), and political (e.g., increasing corruption, rising government instability, weak legal system) risks. Additionally, it is consistent with Kastrati (2011), who established that increasing political stability through government commitment to the rule of law has a vital role in explaining NPLs so that countries with less efficient judicial systems are likely to have a higher rate of NPLs. Particularly, the results reveal that the country risk has a progressively negative effect (Q.25 = − 0.105, Q.50 = − 0.131, Q.75 = − 0.153, Q.95 = − 0.175) on NPLs, indicating that an increase in domestic uncertainties (e.g., economic, financial, political risk) has the most positive outstanding effect on the banking sector of countries with a higher degree of NPLs.

Similarly, the estimation results establish that banking sectors’ NPLs are positively impacted by financial market development (DC/GDP) and lending interest rates (LIR). As documented in previous studies, the results indicate that banks which operate in a more developed financial market (e.g., Boudriga et al. 2010; Adebola et al. 2011) and charge a higher lending interest rate (e.g., Adebola et al. 2011; Beck et al. 2015; Rehmana et al. 2020) are more likely to incur higher levels of NPLs. In this environment, there is a need to mitigate NPLs using effective credit risk practices (e.g., collateral enhancement, and credit ratings). Moreover, the results demonstrate that the coefficient of global risk (GR) is statistically significant and positive, indicating that higher global economic policy uncertainty is positively linked with increasing credit risk in the BRICS banking sector. This suggests that GR has a spillover effect on BRICS economies, and rising GR exacerbates NPLs in BRICS banks. Similar previous research (e.g., Athari 2021a, b; Athari and Bahreini 2021) also stress that a rise in global economic policy uncertainty resulted in declining banks’ profitability in other countries. Overall, the results provide evidence that country and global-level variables are significant predictors of NPLs in BRICS countries.

The estimation results of Eq. (3) for quantiles Q.25, Q.50, Q.75, and Q.95 are reported in Table 8. Consistent with the results in Table 7, profitability, capital regulation, liquidity, and income diversification with negative signs and inefficiency with a positive sign significantly impact banking sector credit risk in the BRICS region. Similarly, the results indicate that profitability has a progressively negative effect on credit risk and the extent of the effect is the most prominent in the 0.95 quantile (Q.95 = − 0.643). Moreover, the estimation results support the previous studies and reveal that banks are likely to have higher NPLs by developing the financial market, increasing the lending interest rate, and rising global economic policy uncertainty.

**Table 8:** The impact of country-specific political, economic, and financial risks on credit risk (2004–2020)

Independent variables	Quantile estimated coefficients			
	Q.25	Q.50	Q.75	Q.95
*Banking sector-specific variables*				
Profitability	− 0.333*	− 0.438**	− 0.565**	− 0.643***
	(0.003)	(0.015)	(0.035)	(0.064)
Capital regulation	− 0.030*	− 0.133	− 0.215	− 0.133***
	(0.001)	(0.325)	(0.121)	(0.064)
Liquidity	− 0.037**	− 0.022***	− 0.175***	− 0.024
	(0.038)	(0.062)	(0.077)	(0.257)
Inefficiency	0.058*	0.037*	0.033	0.027**
	(0.002)	(0.000)	(0.235)	(0.032)
Income diversification	− 0.041	− 0.024	− 0.130*	− 0.032*
	(0.342)	(0.254)	(0.003)	(0.001)
*Country and global-level variables*				
Political risk index	− 0.122**	− 0.141*	− 0.163**	− 0.172*
	(0.015)	(0.000)	(0.025)	(0.005)
Economic risk index	− 0.119	− 0.125*	− 0.123*	− 0.115**
	(0.251)	(0.002)	(0.000)	(0.026)
Financial risk index	− 0.071*	− 0.032**	− 0.124	− 0.052*
	(0.000)	(0.047)	(0.422)	(0.001)
Financial market development	0.037*	0.026**	0.143	0.012
	(0.001)	(0.022)	(0.351)	(0.257)
Lending interest rate	0.018	0.024**	0.034***	0.026**
	(0.316)	(0.036)	(0.095)	(0.042)
Global risk	0.005**	0.023***	0.013*	0.023**
	(0.030)	(0.064)	(0.000)	(0.026)
TC dummies	YES	YES	YES	YES

Table 8 shows the effect of the country-specific political, economic, and financial risks on the BRICS banking sector’s credit risk. Profitability is the bank return on assets; Capital regulation is the bank regulatory capital to risk-weighted assets; Liquidity is the bank liquid reserves to bank assets ratio; Inefficiency is the bank cost to income ratio; Income diversification is the bank non-interest income to total income; Country risk index is the ICRG country risk rating; Financial market development is the domestic credit provided by the banking sector to GDP; Lending interest rate is the bank rate that usually meets the short- and medium-term financing needs of the private sector; Global risk is global economic policy uncertainty. TC dummies are time and country dummies. The 25th, 50th, 75th, and 95th percentiles of the NPL rate are reported. Numbers in parentheses for each column denote the *P* values. *, **, and *** denote the significance level at 1%, 5%, and 10%, respectively

Concentrating on the country-specific risks, the estimation results in Table 8 underscore that the coefficients of political, economic, and financial risks are negative and significant, implying that an increasing political, economic, and financial uncertainty of environments is strongly associated with rising banking sectors’ NPLs in BRICS countries. In other words, the results reveal that the changes in banks’ NPLs depend on the vulnerability of BRICS economies to political, economic, and financial risks. This is in line with previous works (Khwaja and Mian 2005; Kastrati 2011; Gjeci and Marinc 2018; Hakimi et al. 2020; Jenkins et al. 2021), the findings of which highlighted that political risk (e.g., by increasing government instability, lack of commitment to the rule of law, high corruption) is an important driver for the increase in banks’ NPLs. Consistently, the results support those in Creane et al. (2006), who concluded that political factors such as weak judicial empowerment, low bureaucracy quality, and poor implementation of credit policies contribute to increasing NPLs. Similarly, Orlando and Pelosi (2020) also suggested that firms’ NPLs can be reduced by improving the quality of bureaucracy, strengthening the judicial system, and enhancing legal enforcement. Additionally, Kastrati (2011) noted that a strong commitment by the government to the rule of law results in banks conducting greater due diligence and making fewer bad loans.

The results also support those of previous studies documenting that increased economic stability (e.g., by increasing GDP growth, reducing inflation) (Quagliariello 2007; Khan et al. 2018; Syed and Tripathi 2020) and financial stability (e.g., reduction in the real effective exchange rate, increasing current account surplus) (Akkoc and Vatansever 2013; Beck et al. 2015; Kuzucu and Kuzucu 2019), in addition to political stability, led to a reduction in NPLs in the banking sector. Recently, Athari (2022c) and Athari and Irani (2022) underscored that an increase in the country’s specific political and economic risks triggers risk-taking behavior in the banking sector internationally, thereby decreasing banking sector stability.

Remarkably, among country-specific risks, the estimation results provide significant evidence for the progressive negative impact of political risk on NPLs (Q.25 = − 0.122, Q.50 = − 0.141, Q.75 = − 0.163, Q.95 = − 0.172). This implies a rise in domestic political instability has the most positive prominent effect on the banking sector of environments with a higher degree of NPLs. In other words, in an environment where the rate of NPLs is high, the banking sector reacts significantly and sensitively to variations in political risks.

## Robustness checks

This study conducted robustness tests for reliability control objectives of the estimated results. First, Eqs. (2) and (3) are estimated using the new alternatives of the “bank return on equity” (ROE) for measuring profitability, “bank capital to total assets” (C/TA) for measuring capital regulation, and “bank overhead costs to total assets” (OC/TA) for measuring inefficiency. The lagged dependent variable and FC dummy, which equals one in 2008 and 2009 and zero otherwise) are added to the estimation models. The robustness estimation results are presented in Tables 9 and 10. The results are consistent, reliable, and related to those stated above and suggest that the NPLs of the banking sector operating in the BRICS region are shaped by the banking sector-specific, country, and global-level determinants. In terms of bank-specific variables, the estimation results reveal significant impacts of profitability, capital regulation, liquidity, inefficiency, and income diversification on the BRICS banking sectors’ NPLs. For country and global-level factors, the country risk index, in particular the political, economic, and financial risks sub-indices, financial market development, lending interest rate, and global risk significantly impact NPLs though the extent of the effect varies across different quantiles. More specifically, the results stress the progressive effect of profitability, country risk index, and political risk sub-index determinants on credit risk. Overall, the BRICS banking sectors’ NPLs increase in response to increases in the country’s vulnerability and global risk.

**Table 9:** Robustness results I

Independent variables	Quantile estimated coefficients				Fixed effects	GMM-SYS
	Q.25	Q.50	Q.75	Q.95	Coefficients	Coefficients
*Banking sector-specific variables*						
Lagged non-performing loan	0.338	0.352	0.446	0.521	0.246	0.245
	(0.155)	(0.334)	(0.256)	(0.417)	(0.226)	(0.333)
Profitability	− 0.282*	− 0.341**	− 0.348**	− 0.447***	− 0.231**	− 0.122*
	(0.001)	(0.032)	(0.025)	(0.063)	(0.021)	(0.000)
Capital regulation	− 0.132*	− 0.043	− 0.153	− 0.244***	− 0.031	− 0.247
	(0.000)	(0.341)	(0.186)	(0.078)	(0.141)	(0.327)
Liquidity	− 0.024	− 0.024	− 0.014**	− 0.013***	− 0.013***	− 0.021**
	(0.332)	(0.426)	(0.034)	(0.063)	(0.056)	(0.025)
Inefficiency	0.027	0.014**	0.033**	0.010	0.021**	0.024***
	(0.122)	(0.017)	(0.031)	(0.335)	(0.032)	(0.063)
Income diversification	− 0.014	− 0.023***	− 0.025	− 0.023*		
	− 0.011		− 0.031*			
	(0.128)	(0.098)	(0.244)	(0.001)	(0.325)	(0.001)
*Country and global-level variables*						
Country risk index	− 0.142*	− 0.221*	− 0.246***	− 0.316*	− 0.142**	− 0.427*
	(0.003)	(0.001)	(0.068)	(0.002)	(0.028)	(0.000)
Financial market development	0.043*	0.032	0.034**	0.023	0.215	0.011***
	(0.002)	(0.124)	(0.023)	(0.252)	(0.302)	(0.078)
Lending interest rate	0.107**	0.124*	0.113	0.113***	0.021***	0.029
	(0.027)	(0.004)	(0.343)	(0.053)	(0.053)	(0.441)
Global risk	0.024***	0.013**	0.033**	0.028***	0.027*	0.033**
	(0.077)	(0.035)	(0.023)	(0.053)	(0.001)	(0.027)
TC dummies	YES	YES	YES	YES	YES	YES
FC dummy	YES	YES	YES	YES	YES	YES
Adj.R^2^	–	–	–	–	0.54	–
CD-test *(p value)*	–	–	–	–	(0.334)	–
AR (2)	–	–	–	–	–	(0.335)
Hansen-test	–	–	–	–	–	(0.541)
Sargan-test	–	–	–	–	–	(0.438)

Table 9 shows the estimation results of Eq. (2) using the Quantile, Fixed effects with the cluster-robust standard error, and GMM-SYS panel estimation with the robust standard error to heteroscedasticity approaches. Profitability is the bank’s return on equity; Capital regulation is the bank’s capital to total assets, and Inefficiency is the bank’s overhead costs to total assets. TC dummies are time and country dummies. FC dummy is the global financial crisis dummy variable which equals one in 2008 and 2009 and zeroes otherwise. The 25th, 50th, 75th, and 95th percentiles of the NPL rate are reported. CD-test stands for a cross-sectional dependence test. AR (2), Hansen, and also Sargan tests stand as serial correlation and over-identification tests, respectively. Numbers in parentheses for each column denote the *P* values. *, **, and *** denote the significance level at 1%, 5%, and 10%, respectively

**Table 10:** Robustness results II

Independent variables	Quantile estimated coefficients				Fixed effects	GMM-SYS
	Q.25	Q.50	Q.75	Q.95	Coefficients	Coefficients
Lagged non-performing loan	0.246	0.426	0.351	0.417	0.313	0.463
	(0.268)	(0.726)	(0.345)	(0.336)	(0.236)	(0.251)
Political risk index	− 0.114*	− 0.133***	− 0.147**	− 0.158**	− 0.074***	− 0.215**
	(0.001)	(0.078)	(0.032)	(0.015)	(0.084)	(0.034)
Economic risk index	− 0.085	− 0.113**	− 0.105	− 0.132***	− 0.117**	− 0.101*
	(0.315)	(0.031)	(0.265)	(0.076)	(0.025)	(0.001)
Financial risk index	− 0.035	− 0.045*	− 0.015***	− 0.024	− 0.023	− 0.002***
	(0.452)	(0.021)	(0.074)	(0.334)	(0.352)	(0.086)
Control variables	YES	YES	YES	YES	YES	YES
TC dummies	YES	YES	YES	YES	YES	YES
FC dummy	YES	YES	YES	YES	YES	YES
Adj.R^2^	–	–	–	–	0.61	–
CD-test *(p value)*	–	–	–	–	(0.287)	–
AR (2)	–	–	–	–	–	(0.246)
Hansen-test	–	–	–	–	–	(0.433)
Sargan-test	–	–	–	–	–	(0.359)

Table 10 shows the estimation results of Eq. (3) using the Quantile, Fixed effects with the cluster-robust standard error, and GMM-SYS panel estimation with the robust standard error to heteroscedasticity approaches. Profitability is the bank’s return on equity; Capital regulation is the bank’s capital to total assets, and Inefficiency is the bank’s overhead costs to total assets. TC dummies are time and country dummies. FC dummy is the global financial crisis dummy variable which equals one in 2008 and 2009 and zeroes otherwise. The 25th, 50th, 75th, and 95th percentiles of the NPL rate are reported. CD-test stands for a cross-sectional dependence test. AR (2), Hansen, and also Sargan tests stand as serial correlation and over-identification tests, respectively. Numbers in parentheses for each column denote the *P* values. *, **, and *** denote the significance level at 1%, 5%, and 10%, respectively

Second, to check the consistency of the results and control the endogeneity problem, we estimate Eqs. (2) and (3) by performing both the fixed effects by clustering standard errors^9^ and GMM-SYS dynamic panel data approach with the robust standard error to heteroscedasticity. Notably, Baltagi et al. (2010) and Newey and West (2014) argued that the fixed effects with the cluster-robust standard error are robust to heteroskedasticity and serial correlation in the error term. Similarly, there is a likelihood of endogeneity problems between dependent and independent variables in the estimation models. Hence, we assume that the independent variables are endogenous. In testing the validity of the estimated models via the fixed effects and GMM-SYS approach, the cross-sectional dependence (Pesaran 2004), serial correlation (AR (2)), and over-identification check (Hansen and Sargan tests)^10^ are applied (e.g.,Athari and Irani 2022; Patra and Padhi 2022). Consistently, as reported in Tables 9 and 10, the estimation results by employing the fixed effects and GMM-SYS approaches reveal that the credit risk of the banking sector in BRICS countries is significantly impacted by the using factors.

Third, this study performs the Granger causality test to examine the linkage direction between the studied factors. We use this test to determine if there is an inverse association between the dependent and independent variables to avoid endogeneity problems. As presented in Table 11, there is statistically significant evidence of Granger causality from the set of independent variables (liquidity, capital regulation, profitability, inefficiency, income diversification, country risk index, political risk index, economic risk index, financial risk index, financial development, lending interest rate, global risk) to NPLs in the panel countries. This implies the estimation model is less likely to suffer from the endogeneity issue and historical information of the explanatory factors can suggest future information about banking sectors’ credit risk in the panel of BRICS countries.

**Table 11:** Robustness results III

Null hypothesis	F-statistics	[Prob. value]	Granger causality
Liquidity → Non-performing loans	3.326*	[0.001]	Yes
Capital regulation → Non-performing loans	4.415*	[0.000]	Yes
Profitability → Non-performing loans	3.456*	[0.003]	Yes
Inefficiency → Non-performing loans	2.426**	[0.024]	Yes
Income diversification → Non-performing loans	4.326*	[0.001]	Yes
Country risk index → Non-performing loans	5.411**	[0.013]	Yes
Political risk index → Non-performing loans	4.165*	[0.001]	Yes
Economic risk index → Non-performing loans	4.353*	[0.000]	Yes
Financial risk index → Non-performing loans	3.435**	[0.032]	Yes
Financial development → Non-performing loans	4.465*	[0.003]	Yes
Lending interest rate → Non-performing loans	5.233*	[0.000]	Yes
Global risk → Non-performing loans	4.336*	[0.001]	Yes

* and **Denote 1% and 5% statistical significance levels, correspondingly

## Conclusion and policy implications

While numerous empirical studies have investigated the credit risk in the banking sector in developed and emerging markets, the determinants of the banking sector’s credit risk in the BRICS emerging economies has received much less consideration. More specifically, limited studies extensively examine the country risk impact, in particular political, economic, and financial risks on the banking sector’s credit risk. Thus, this study fills the gap in the literature by probing the effects of the country risk index and the country’s sub-indices such as the economic, political, and financial risks level on the BRICS banking sectors’ NPLs. To do so, panel data analysis is performed using the quantile estimation approach between the wide range from 2004 to 2020.

The findings demonstrate that profitability, capital regulation, liquidity, and income diversification determinants with negative signs, but inefficiency with a positive sign significantly impact credit risk in the BRICS banking sector. Moreover, the results reveal that the country risk has a negative and statistically significant effect on the credit risk, implying that the sectors’ NPLs are positively associated with countries’ rising vulnerability to domestic risk, and this effect is prominent in the banking sector of countries with a higher degree of NPLs. More specifically, the result underscores that the coefficients of political, economic, and financial risks are negative and significant, implying that increasing political, economic, and financial uncertainty of environments is strongly associated with rising credit risk. Among country-specific risks, the estimation results imply that a rise in domestic political instability, in particular, has the most positive prominent effect on the banking sector of countries with a higher degree of credit risk. Moreover, the estimation results indicate that the BRICS banking sector’s credit risk is positively impacted by the developing financial markets, increasing the lending interest rate, and soaring global risk.

Results have significant implications for policymakers, regulators, bankers, and analysts. First, the results suggest that by preparing a BRICS environment less vulnerable to domestic risks, policymakers and regulators can reduce NPLs in the banking sector, thereby limiting the negative impact of credit risk (e.g., increasing banking instability) and on the contrary increase economic performance. Banking sector instability has negative effects on financial market stability and real sector output, resulting in less efficient resource allocation, increased financial instability, and increased uncertainty regarding future output growth. Second, the results particularly suggest that policymakers and regulators should be aware of the vulnerability of the BRICS environment to specific economic, financial, and political risks to reduce credit risk in the banking sector. This could be possible by considering the related components with the economic risk (e.g., reducing inflation, increasing GDP), financial risk (e.g., reducing exchange rate volatility), and political risk (e.g., reducing corruption, reducing government instability, reducing internal and external conflicts, implementing the rule of law).

Third, policymakers and bankers should pay more attention to important determinants specific to the banking sector and help reduce NPLs by improving profitability, capital regulation, liquidity, income diversification, and efficiency. Fourth, as financial markets develop and lending interest rates rise, policymakers and bankers must employ effective practices (e.g., increasing collateral, and credit ratings) to significantly reduce credit risk. Fifth, the results underscore the need for bank managers to develop a useful framework aimed at maintaining financial stability through income diversification, improving efficiency, and adopting prudent credit risk management to prevent the severe impact of global risks on NPLs in the banking sector.

Although this study provides strong empirical results for modeling the impact of country risk, especially financial, economic, and political risks, on the banking sector’s credit risk of BRICS countries, further empirical research should investigate this nexus for other regions such as MENA or MINT countries to provide a comprehensive picture.

## Data Availability

The datasets used and/or analyzed during the current study are available from the corresponding author on reasonable request.

## Abbreviations

BRICS: Brazil, Russia, India, China, and South Africa
NPLs: Non-performing loans
BCBS: Basel Committee on Banking Supervision
GDP: Gross domestic production
MENA: Middle East and North Africa
GCC: Gulf Corporation Council
MINT: Mexico, Indonesia, Nigeria, Turkey
SAARC: South Asian Association for Regional Cooperation
ICRG: International Country Risk Guide

## Footnotes

1 Kou et al. (2021b) by using transactional data and two-stage multiobjective feature selection predicted bankruptcy for SMEs, which eventually helps to reduce banks’ NPLs.

2 Kou et al. (2021a) showed that Fintech-based investment alternatives help improve the financial performance of European banks.

3 Li et al. (2021) developed an integrated approach to detect clusters in large-scale financial datasets.

4 Kou et al. (2014) evaluated the clustering algorithms for financial risk analysis using MCDM methods.

5 www.policyuncertainty.com.

6 A higher country risk index score indicates a lower country’s vulnerability.

7 A higher global economic policy uncertainty index score indicates a higher global risk.

8 Further information is available at: www.prsgroup.com.

9 The fixed effects model with Driscoll Kraay standard error is also adopted as a robustness test for the possibility of having cross-sectional and heteroscedasticity. Consistently, the results are similar and for the sake of brevity, we do not report it.

10 The Hansen statistic is a test of over-identifying restrictions, asymptotically distributed as × 2(k) under the null of valid instruments. Similarly, the null hypothesis of the Sargan test is overidentifying restrictions are valid, and the model can be used for analysis.
